# Deicing Concrete Pavements and Roads with Carbon Nanotubes (CNTs) as Heating Elements

**DOI:** 10.3390/ma13112504

**Published:** 2020-05-30

**Authors:** Hee Su Kim, Hoki Ban, Won-Jun Park

**Affiliations:** 1 Department of Civil Engineering, Kangwon National University, Samcheok 25913, Korea; teriary@kangwon.ac.kr; 2 Department of Architectural Engineering, Kangwon National University, Samcheok 25913, Korea

**Keywords:** carbon nanotubes, heating element, concrete beam, deicing technology

## Abstract

Existing deicing technologies involving chloride and heating wires have limitations such as reduced durability of roads and surrounding structures, and high labor requirements and maintenance costs. Hence, in this study, we performed indoor experiments, numerical analyses, and field tests to examine the efficiency of deicing using carbon nanotubes (CNTs) to overcome these limitations. For indoor experiments, a CNT was inserted into the center of a concrete sample and then heated to 60 °C while maintaining the ambient and internal temperatures of the sample at −10 °C using a refrigeration chamber. Numerical analysis considering thermal conductivity was performed based on the indoor experimental results. Using the calculation results, field tests were conducted, and the thermal conduction performance of the heating element was examined. Results showed that the surface temperature between the heating elements exceeded 0 °C. Moreover, we found that the effective heating distance of the heating elements should be 20–30 cm for effective thermal overlap through the indoor experiments. Additionally, the numerical analysis results indicated that the effective heating distance increased to 100 cm when the heating element temperature and experiment time were increased. Field test results showed that 62 cm-deep snow melted between the heating elements (100 cm), thus, verifying the possibility of deicing.

## 1. Introduction

Accidents caused by black ice in winter are increasing every year. Icing on roads in winter decreases the friction between the road surface and vehicles, causing major traffic accidents with a high fatality rate [1,2]. The “Characteristics of and Countermeasures to Black Ice Traffic Accidents in Winter” published by Samsung Traffic Safety Research Institute [3] reports that 6548 traffic accidents and 199 fatalities occurred in South Korea in the past five years, with a fatality rate of 3.0 deaths per 100 accidents. The fatality rate of traffic accidents in winter is 1.6 times higher than the fatality rate during all traffic accidents. Hence, to prevent these accidents caused by black ice, deicing technologies are being researched and applied; some methods include using deicing chemicals (sodium chloride or calcium chloride), installation of heating wires and geothermal pipes, and deicing based on solar heat. Hossin et al. [4,5] studied the effectiveness of road salt with different pavement types using field tests. Wang et al. [6] and Kim et al. [7] reported that although deicing chemicals are widely used because they are inexpensive and easy to store, they could damage road pavements and bridges. Moreover, deicing chemicals are major contributors to environmental pollution [8,9,10]. Therefore, to prevent damage due to deicing chemicals, Caddet [11], Lee et al. [12], and Choi and Hwang [13] researched deicing technologies using geothermal pipes installed underneath roads. However, geothermal deicing offers low deicing efficiency during snow because it uses geothermal heat at a certain temperature. Furthermore, geothermal deicing requires large areas for installing turbines and it is difficult to install them in mountains. To overcome these drawbacks, deicing technologies using heating wires and electrically conductive concrete offering excellent deicing performance are being researched. Chang et al. [14] attached carbon nanofibers to the bottom of a concrete sample and the surface temperature rose to 5 °C at an ambient temperature of −12 °C. Suh et al. [15] installed underground heating mesh at 5 and 10 cm from the surface of a concrete sample, and the surface temperature reached 0–8 °C at an ambient temperature of −18 to −2 °C. Zhao et al. [16] and Wu et al. [17] installed carbon fiber heating wires at a spacing of 10 cm in a concrete sample and reported that the surface temperature increased to 6–8 °C. Lai et al. [18] installed carbon fibers spaced at 10 and 15 cm in a concrete sample and the surface temperature exceeded 0 °C at an ambient temperature of −5 °C. Thus, previous works on deicing technologies using heating wires show excellent deicing effects, but they are also associated with problems such as re-pavement when used on existing roads.

Therefore, our study aimed to develop a deicing technology applicable to existing roads as well as new roads through conducting indoor experiments, numerical analyses, and field tests. For the indoor experiments, we inserted a heating element, namely a carbon nanotube (CNT) in a concrete sample and determined the effective heating distance (distance from the heating element to the point where the surface temperature is 0 °C). In addition, we inserted two heating elements into samples spaced at 15, 20, and 30 cm and verified the increase in the surface temperature, owing to the thermal overlap effect. Further to this, we analyzed the indoor experimental results numerically. Based on these results, the thermal overlap effect according to the distance was verified by increasing the distances between the heating elements to 100, 150, and 200 cm. Field tests were performed to verify the numerical analysis results by installing heating elements spaced at 1 m, arranged in an inverted S-shaped layout at a concrete test site measuring 300 × 300 × 10 cm.

## 2. Indoor Experiments and Results

### 2.1. Fabricating Concrete Samples

For the indoor experiments, concrete samples were fabricated according to the mixing following proportions given in the concrete pavement design standard of the Korea Expressway Corporation for concrete pavements: cement (class 1, 3.15), fine aggregate (2.60), and coarse aggregate (2.65). Because their specific gravities are different from those of the cement (class 1, 3.15), fine aggregate (2.66), and coarse aggregate (2.73) used in this study, we performed concrete mixing after correction. Furthermore, although air-entraining agents and superplasticizers were not included, they were added at a ratio of 0.6% cement to the concrete pavement slump value after mixing and for convenience of work. Table 1 provides the mixing proportion design used in this study.

**Figure 1:**
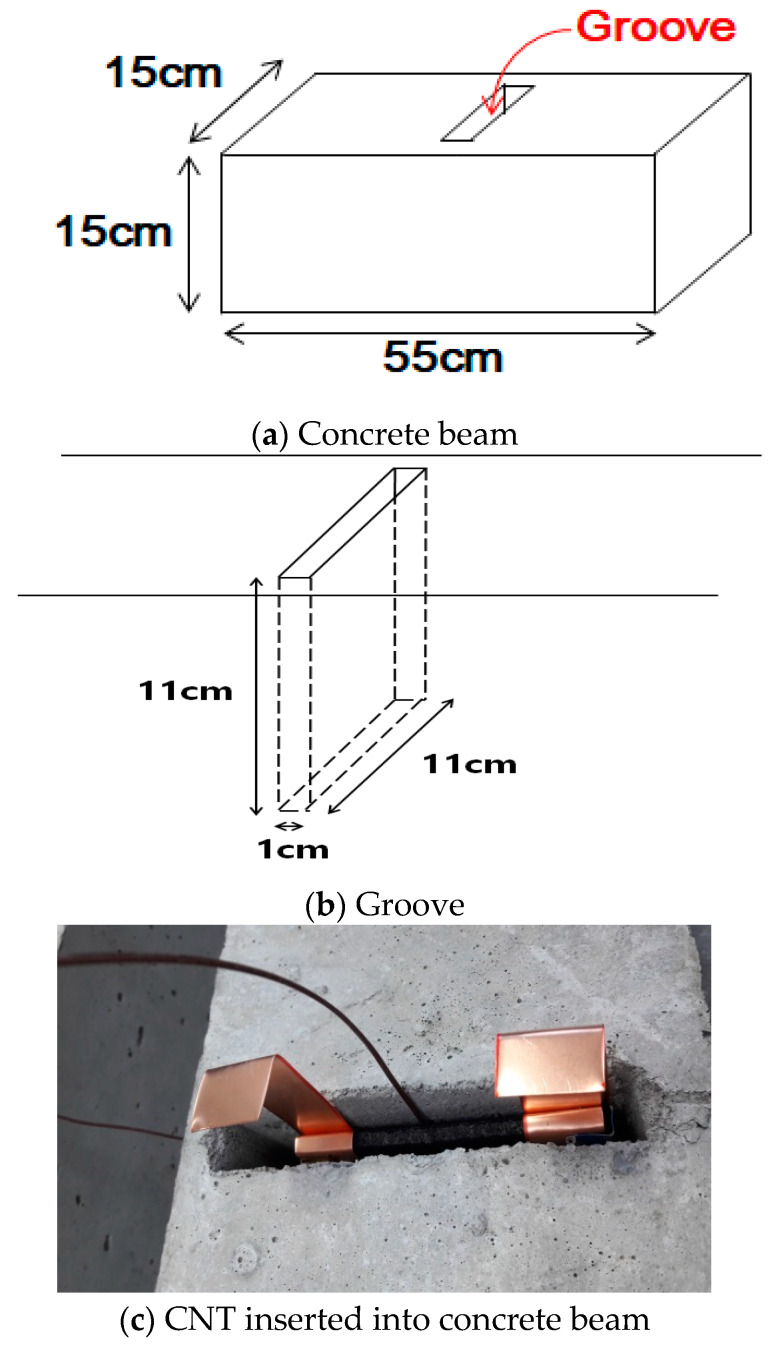
Schematic of concrete beam.

To develop a deicing method applicable to existing roads, we created a groove in the concrete and inserted the heating element into it (Figure 1). The groove was formed by inserting a plywood measuring 11 × 11 × 1 cm at the center of the sample with a size of 55 × 15 × 15 cm and then, wet curing was performed for seven days. After removing the plywood from the wet-cured sample, the heating element (CNTs in this study) measuring 10 × 10 × 0.5 cm was inserted into the groove and the empty part was filled with mortar and then wet cured for seven days. The CNTs properties listed in Table 2 were used.

### 2.2. Experiments

**Figure 2:**
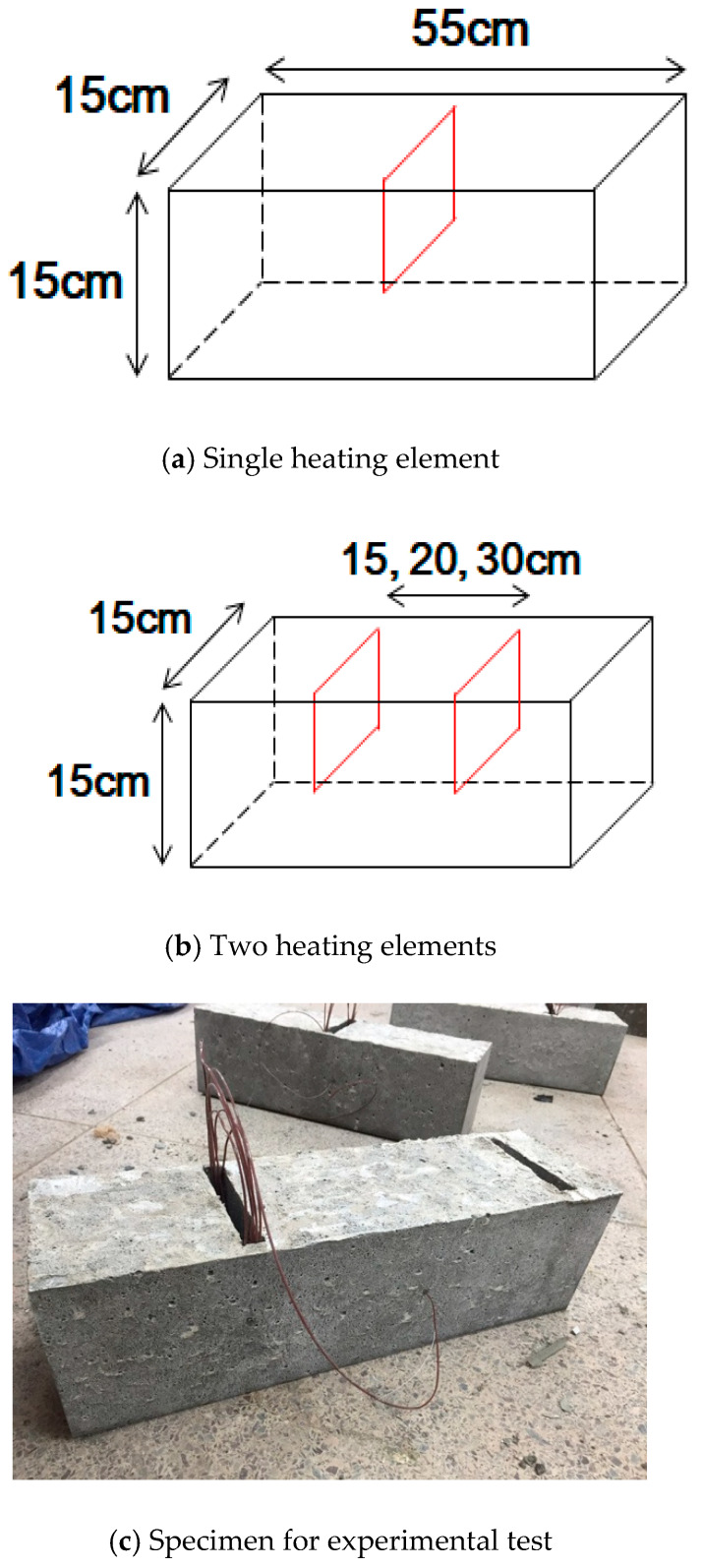
Schematic showing heating elements embedded in concrete beam.

To examine the thermal conductivity effect of the heating elements (CNTs in this study, CNT Solution, Pyeongtaek, South Korea) and the overlap effect between the heating elements, eight samples considering two cases were fabricated. In the first case, one heating element was embedded in the center of the concrete sample, as shown in Figure 2a. In the second case, two heating elements spaced at 15, 20, and 30 cm were embedded, as shown in Figure 2b. For the indoor experiment, the temperature at the sample center was lowered to −10 °C using a refrigeration chamber that can maintain ambient temperatures up to −10 °C; then, the heating element temperature was maintained at 60 °C by operating the heating element. The experiment was performed for 2 h, and the concrete surface temperature, center temperature, and ambient temperature of the sample were measured every minute.

### 2.3. Results

**Figure 3:**
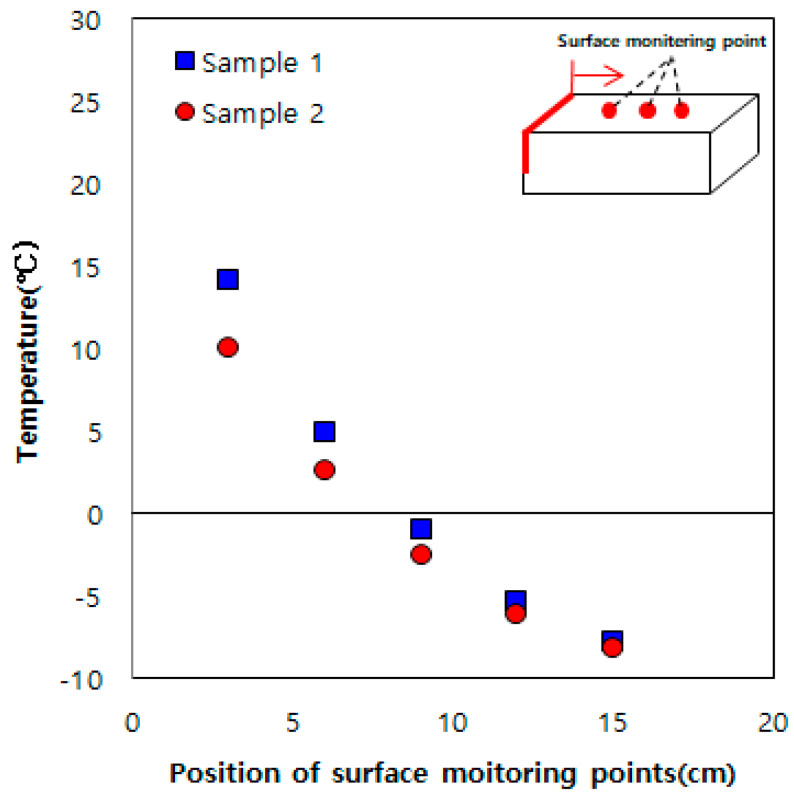
Results obtained using a single heating element.

Figure 3 shows the experiment results for the sample embedded with one heating element. The average effective heating distance (distance from the heating element to the point where surface temperature is 0 °C) was 8 cm. Furthermore, the average temperature at 15 cm from the heating element was −7.5 °C, higher by 2.5 °C than the initial sample temperature and ambient temperature (−10 °C). Therefore, it was inferred that the heat generated by the heating element was transmitted to the entire concrete sample.

**Figure 4:**
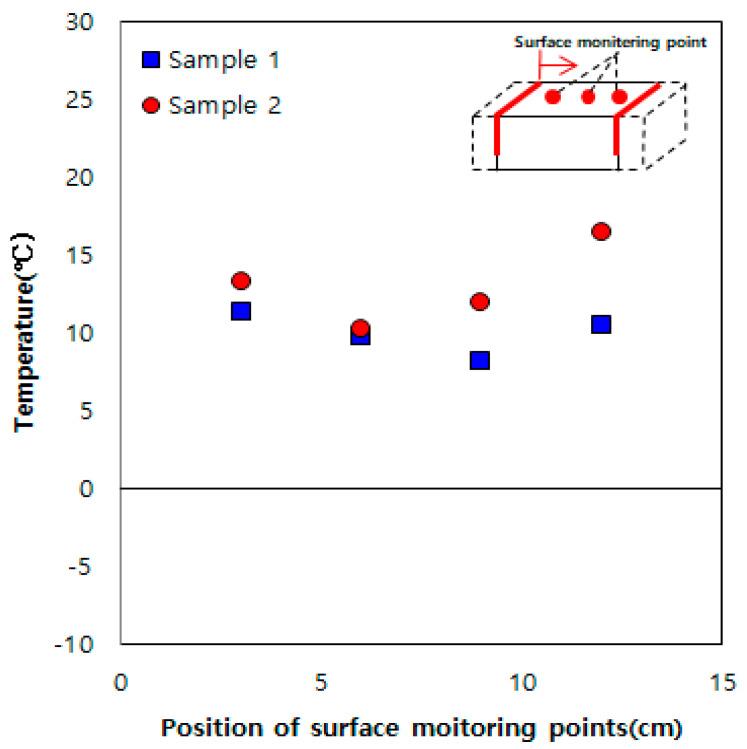
Results obtained using two heating elements spaced at 15 cm.

Figure 4 shows the experimental results for the sample embedded with two heating elements at a spacing of 15 cm. Heat is generated by these two heating elements, and they are symmetrical to each other. Unlike the result obtained using one heating element (Figure 3), the entire section between the heating elements shows a temperature higher than 0 °C, owing to the thermal overlap effect between the heating elements.

**Figure 5:**
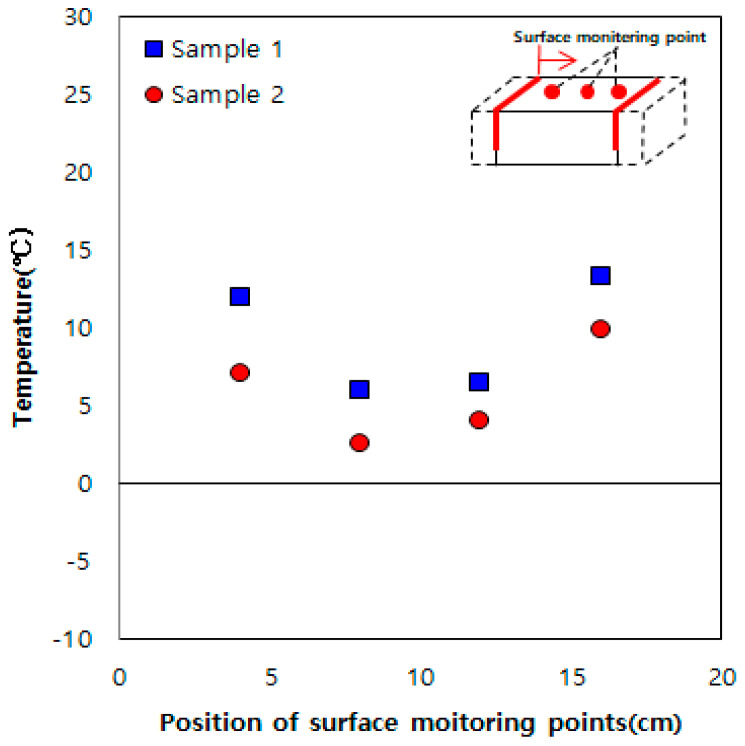
Results obtained using two heating elements spaced at 20 cm.

**Figure 6:**
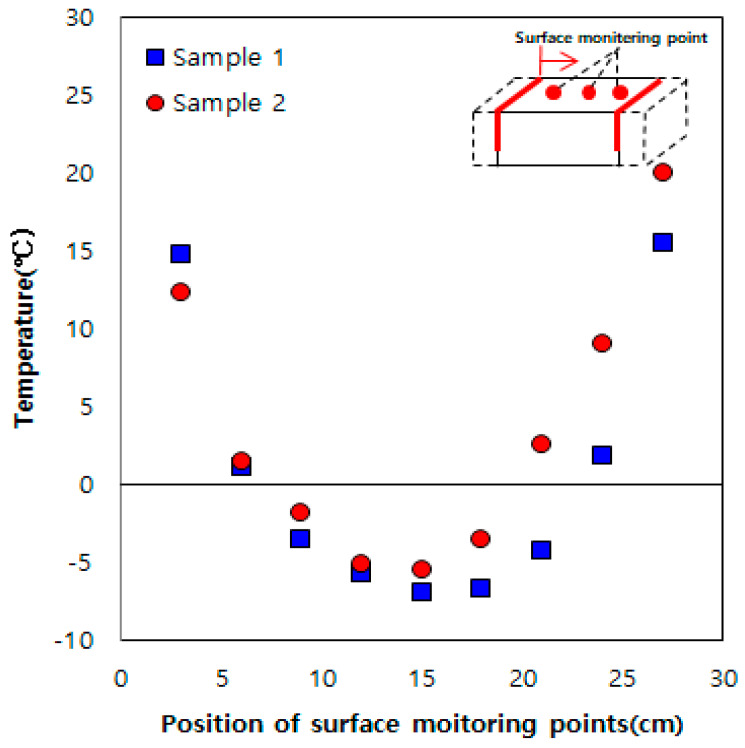
Results obtained using heating elements spaced at 30 cm.

To examine the effective distance of the thermal overlap effect, experiments were performed with distances of 20 and 30 cm between the heating elements and the results are shown in Figure 5 and Figure 6. At a spacing of 20 cm, the rise in surface temperature was lower compared to that observed for a spacing of 15 cm, but the temperature was still higher than 0 °C. In contrast, for a spacing of 30 cm, the temperature fell below 0 °C near the center of the two heating elements (15 cm from each heating element). However, the average surface temperature at the center between the two heating elements was −5 °C; this was 2.5 °C higher than that observed when using one heating element at the same spacing. Hence, at a spacing of 30 cm, although thermal overlapping was not effective (surface temperature higher than 0 °C) owing to the large space between the two heating elements, heat was transmitted to the entire sample. Therefore, we determined the effective heating distance of the heating elements to be 20–30 cm through this indoor experiment.

## 3. Numerical Analysis

### 3.1. Heat Transfer Analysis

The heat transfer process between the concrete sample and heating element is very complex. However, for the numerical analysis model used in this study, we assumed that the mechanical properties as well as the isotropy of the material did not change with temperature. The governing equation for heat transfer analysis can be expressed as follows [19]:

(1) $$\rho c\frac{\partial T}{\partial t}=k_{x}\frac{\partial^{2}T}{\partial x^{2}}+k_{y}\frac{\partial^{2}T}{\partial y^{2}}+k_{z}\frac{\partial^{2}T}{\partial z^{2}}+q$$

where ρ: density;

T: temperature;

C: specific heat;

$k_{x},\;k_{y},\;\mathrm{and}\;k_{z}$: thermal conductivities in the x, y, and z directions;

q: heating value.

The thermal conductivity was assumed identical in every direction. 

### 3.2. Simulations and Results

To verify the indoor experimental results, the heat transfer process was simulated, including the thermal conductivity, specific heat, film coefficient (heat transfer speed through the upper unit area of the transfer surface), and heat flux. 

**Figure 7:**
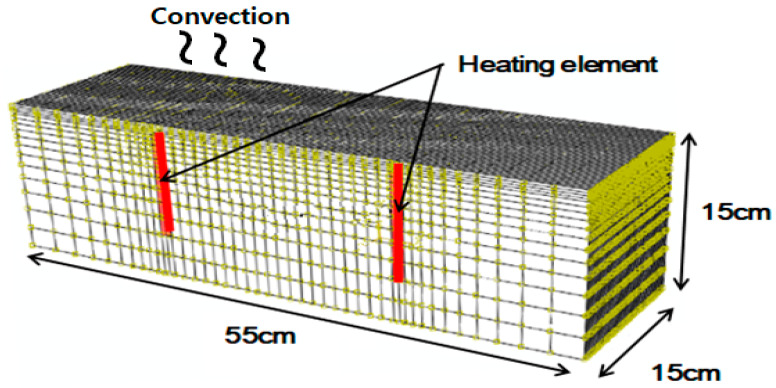
Element mesh and boundary conditions used for the simulations (laboratory tests).

For the simulations, the commercial software package ABAQUS was used [1]. Figure 7 shows the finite element mesh and boundary conditions used for the simulations. The side and bottom of the concrete are fully insulated, and natural convection is applied to the surface.

We used the film coefficient for the heat transfer between the concrete beam and air was estimated using the following empirical equation of Jürges [20]:

(2) $$h_{a}=\{\begin{array}{c} 3.95u_{a}+5.58\;\left(u_{a}\leq 4.8\;m/s\right)\\ 7.14u_{a}{}_{}^{0.78}(u_{a}>4.8\;m/s) \end{array},$$

where $h_{a}$: film coefficient for heat transfer between concrete beam and air;

$u_{a}$: wind speed.

Because the indoor experiments were performed using a refrigeration chamber ($u_{a}=0$), the film coefficient ($h_{a}$) was simply calculated as $5.58{\;\mathrm{Wm}}^{2}/K$. The parameters (properties) required for heat transfer using one heating element were determined through repeated analyses and a matching process with the experimental results. Then, these parameters were verified through comparison with the experimental results obtained for heating elements installed at distances of 15, 20, and 30 cm. The concrete properties listed in Table 3 were used.

**Figure 8:**
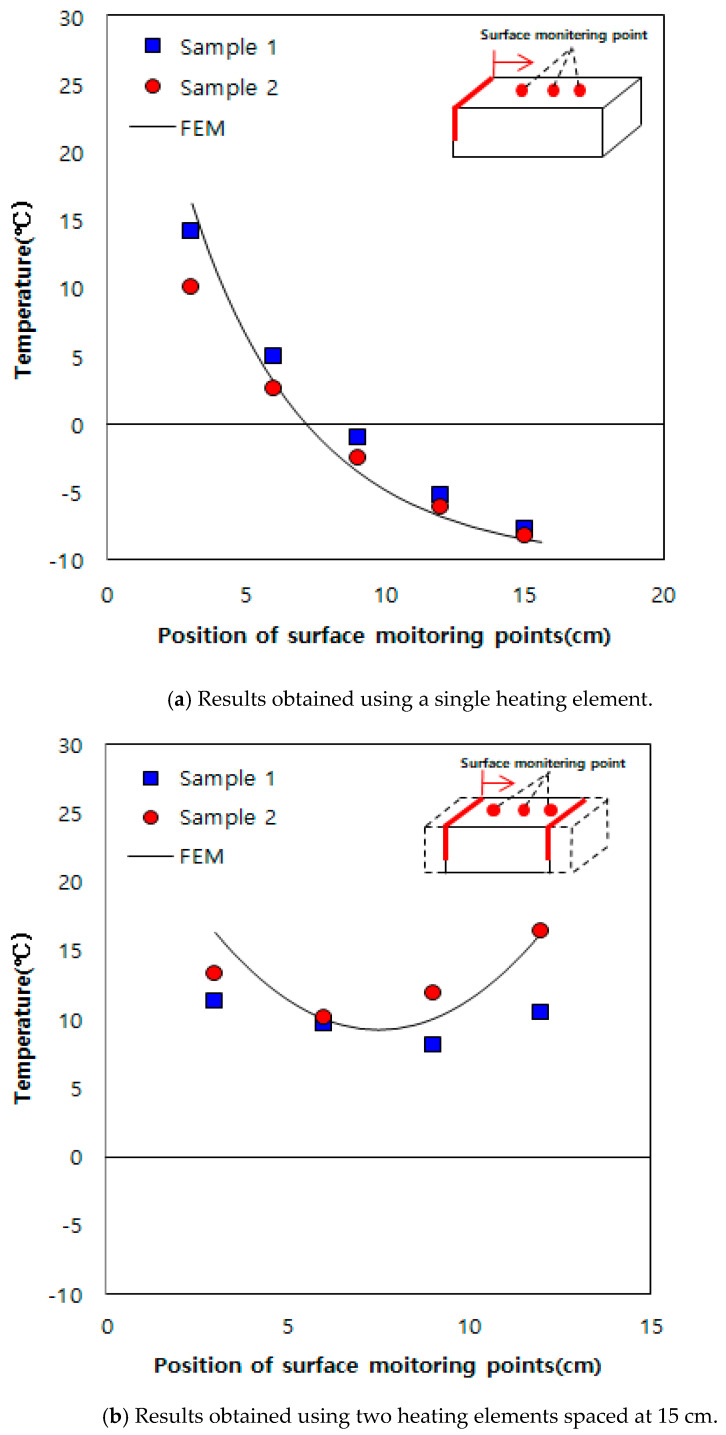

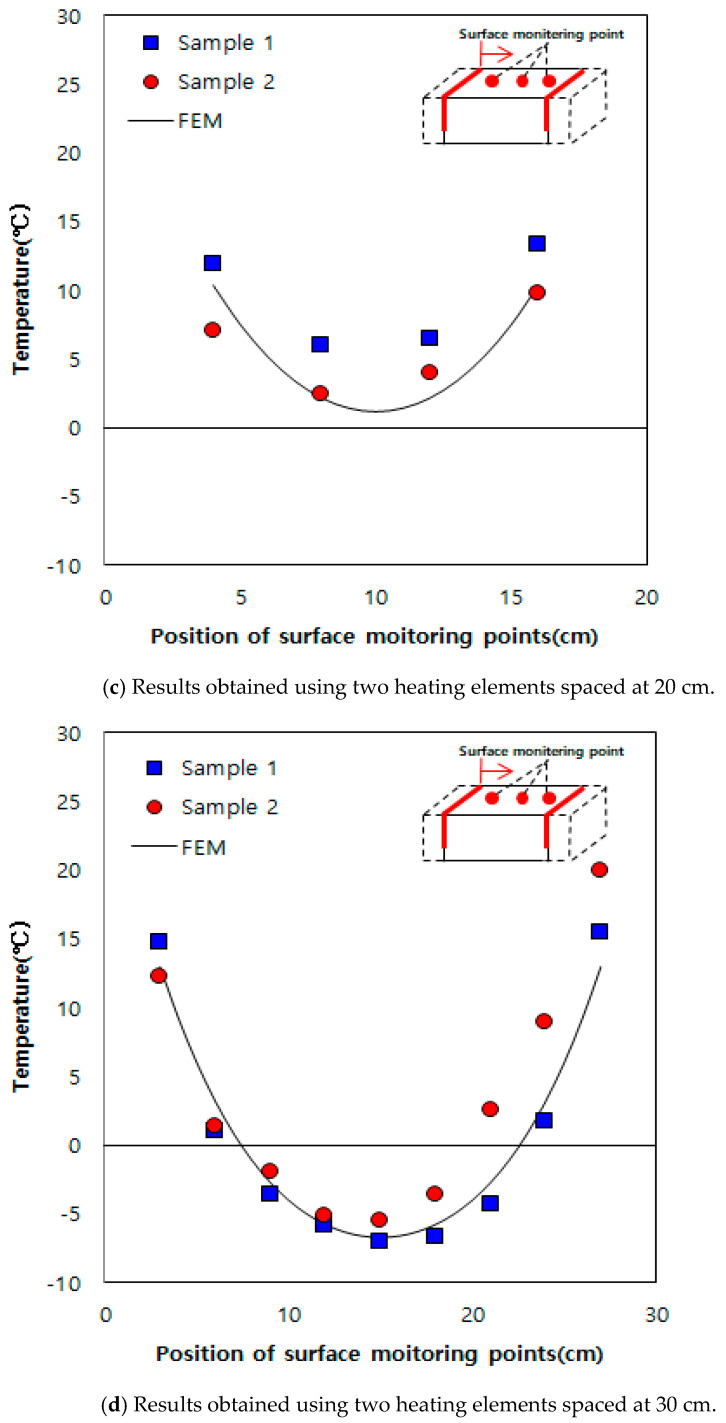
Comparisons between numerical simulations and laboratory tests.

The following were the properties used for obtaining the results shown in Figure 8 through the matching process with the experiment results: thermal conductivity of 1.1 W/m·°C, specific heat capacity of 750 J/kg·°C, and film coefficient of $5.58{\;\mathrm{Wm}}^{2}/K$.

**Figure 9:**
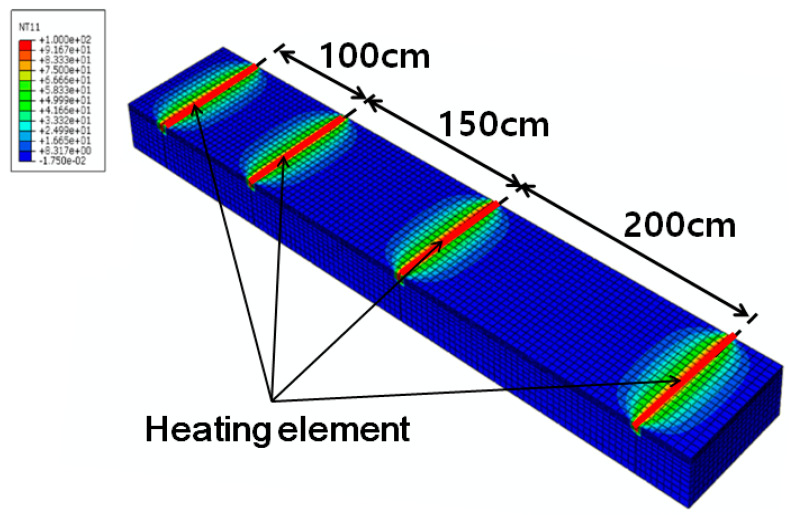
Results of numerical simulations for the field tests.

**Figure 10:**
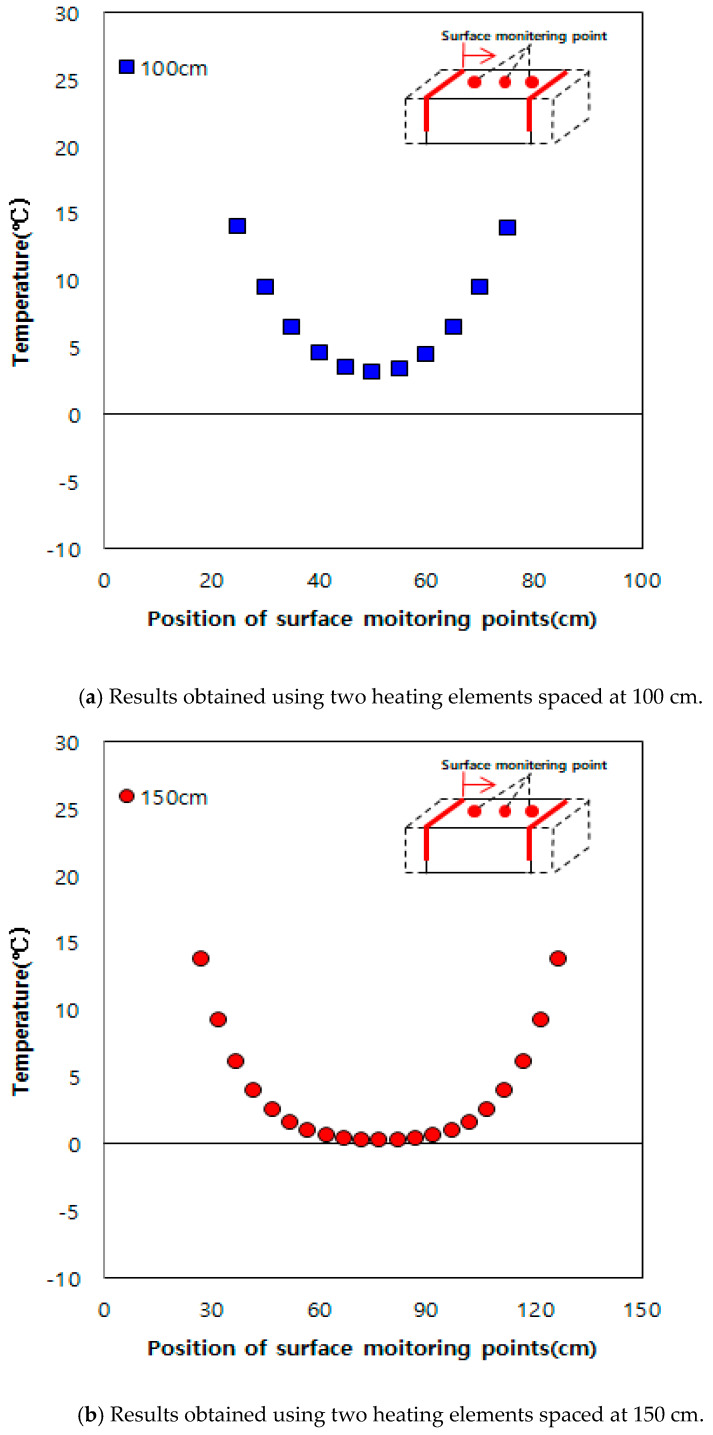

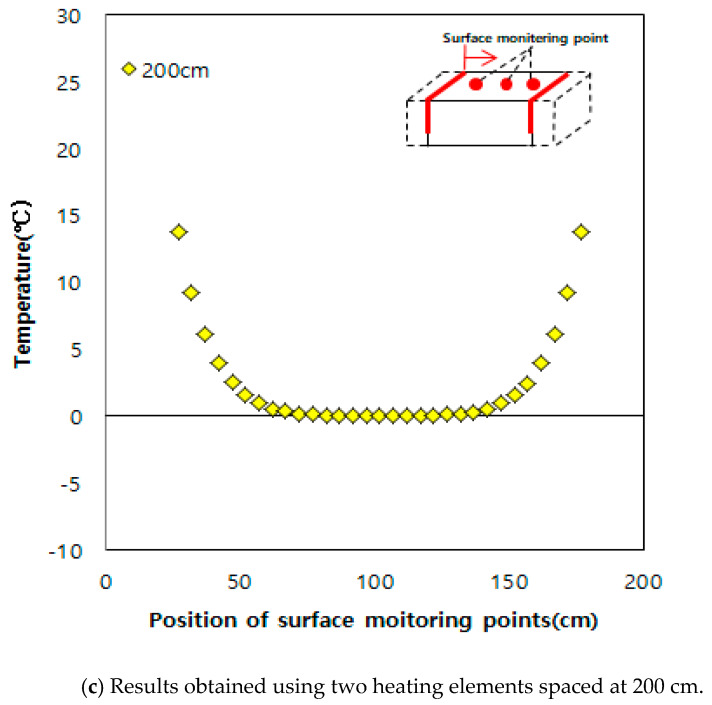
Numerical analysis results.

When grooves are formed at a spacing of 20 cm on an existing road in service and heating elements are inserted into them, there are concerns regarding strength degradation and damage of the road pavement. Therefore, numerical analysis was conducted through inserting heating elements spaced at 100, 150, and 200 cm, as shown in Figure 9, using the previously obtained thermal properties of concrete. For the indoor experiments, owing to the small sizes of the samples and heating elements, we performed the experiments for 2 h using a heating element temperature of 60 °C. However, for numerical analysis, a heating element temperature of 100 °C and heating time of 3 h were used to apply the developed deicing technology practically. Figure 10 shows the numerical analysis results. At a spacing of 100 cm shown in Figure 10a, the surface temperature between the heating elements was 3 °C. However, when the distances were 150 and 200 cm, as shown in Figure 10b,c, the surface temperature was approximately 0 °C and below 0 °C, respectively. Therefore, based on the numerical analysis results, it was determined that the effective heating distance was 100 cm.

## 4. Field Tests and Results

### 4.1. Test Procedures

**Figure 11:**
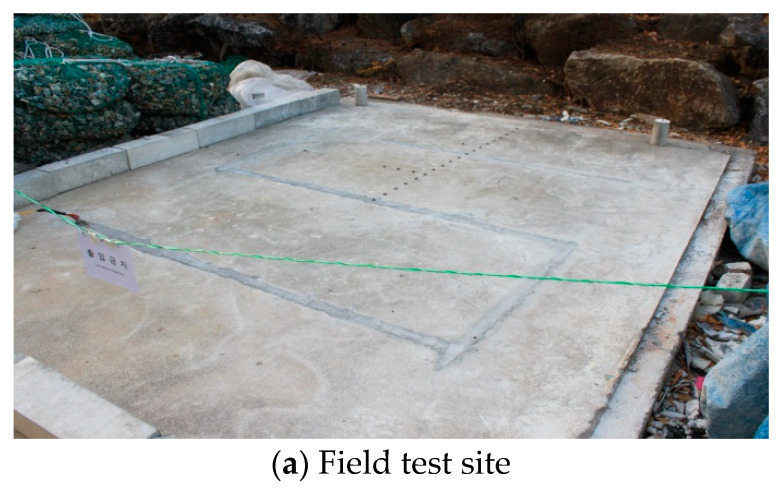

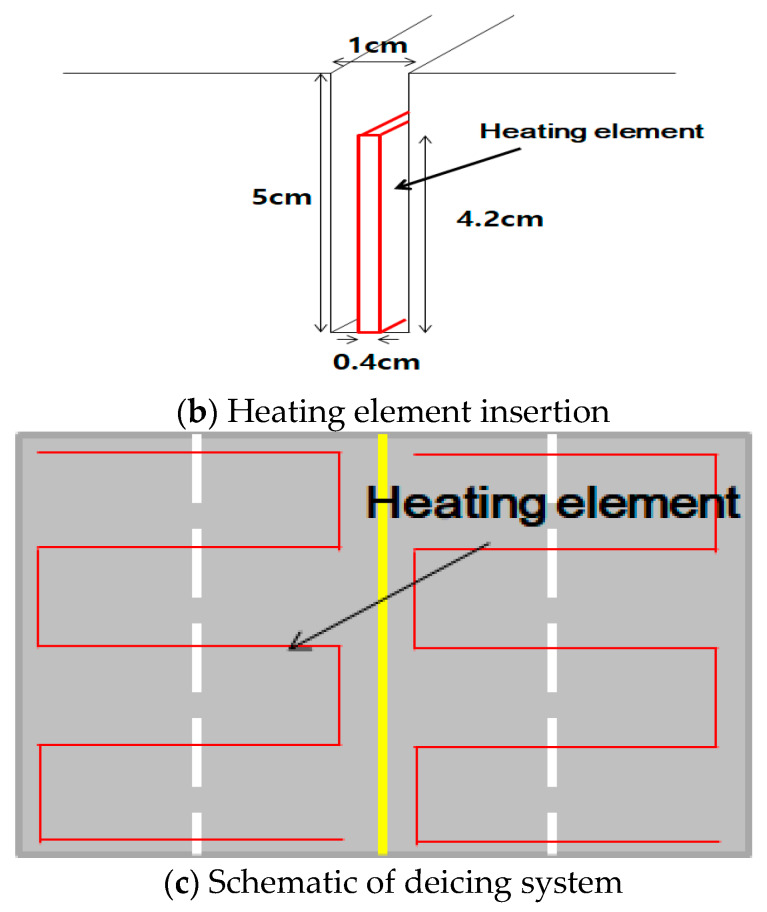
Overview of field test procedure.

Field tests were performed to determine the possibility of deicing by applying the deicing technology developed in this study on an existing road in service. Four field tests were performed, including three tests to verify the change in surface temperature due to thermal overlap and one test for verifying the possibility of deicing by melting snow. For the field tests, 1 × 5 cm grooves with a length of 800 cm were formed, as shown in Figure 11b, in a concrete slab measuring 300 × 300 × 10 cm (Figure 11a). Heating elements with dimensions of 0.4 × 4.2 × 800 cm were inserted into the grooves, and the empty parts were filled with mortar. Then, the samples were wet cured for seven days. As shown in Figure 11c, the heating elements are arranged in an inverted S-shaped layout in the field tests as well. The same mixing proportions for concrete and mortar used in the indoor experiments were used for sample preparation in the field tests.

**Figure 12:**
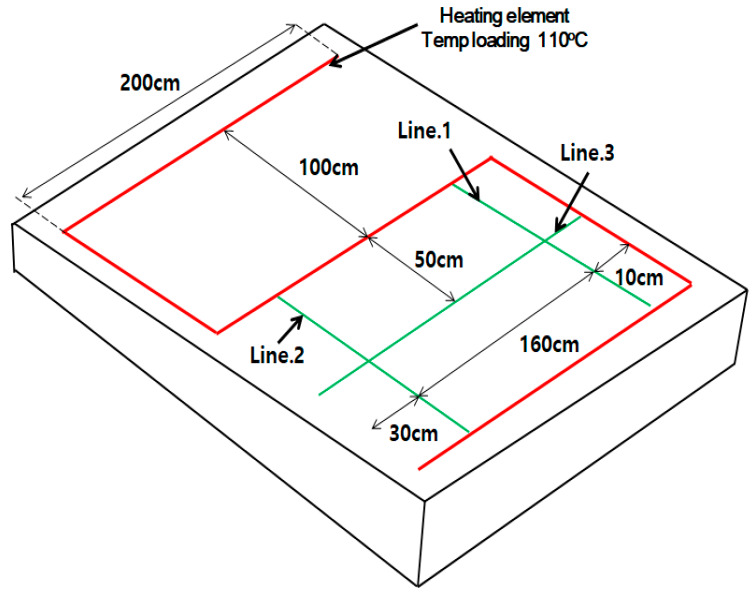
Temperature measurement locations.

The changes in surface temperatures during field tests were measured using iButton temperature sensors (Table 4). The iButtons were attached to lines 1, 2, and 3 shown in Figure 12. Line 1 was expected to show the highest thermal overlap effect of the heating elements, and line 2 was expected to show the smallest thermal overlap effect. Furthermore, in line 3, we verified the temperature change at the center between heating elements. For continuity, the iButtons were spaced at 10 cm. This field test was performed for 12 h while maintaining the heating element temperature at 110 °C.

### 4.2. Results

**Figure 13:**
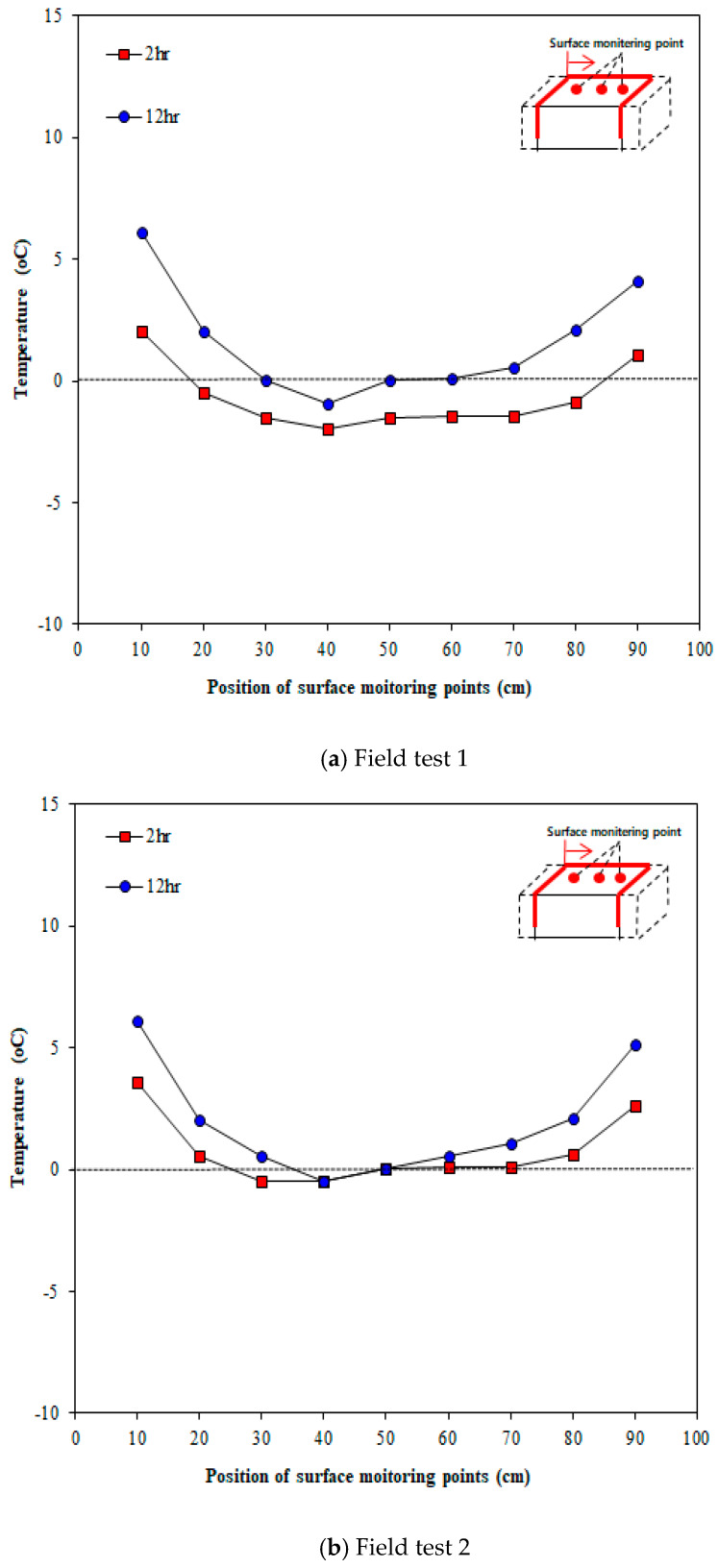

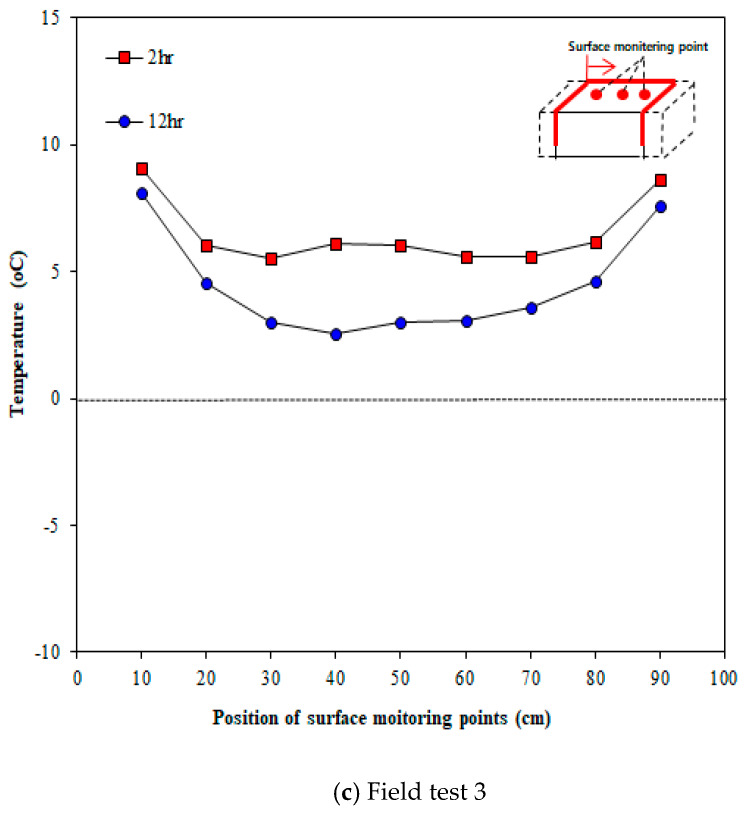
Temperatures at different surface locations and measurement times (Line 1).

**Figure 14:**
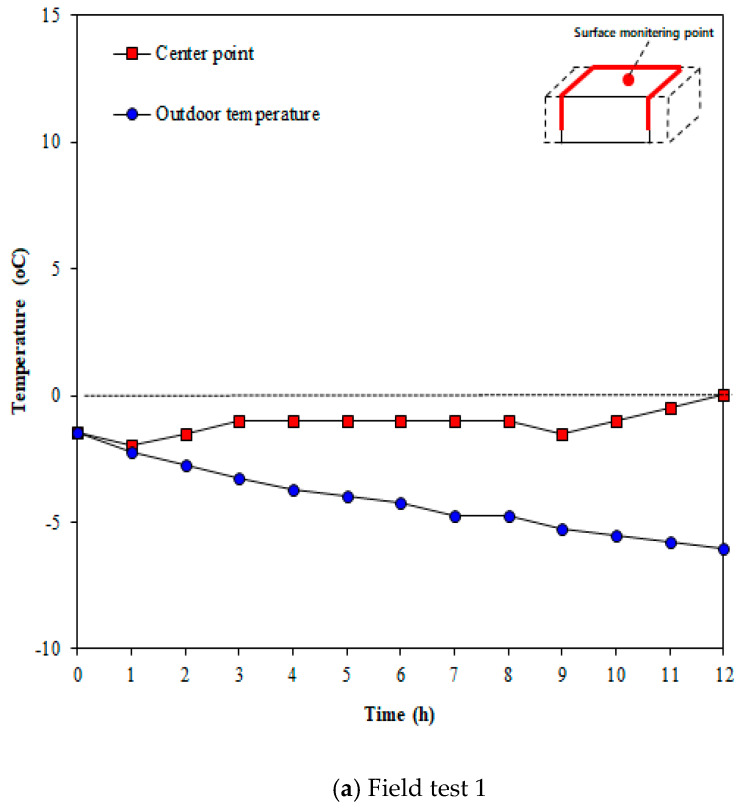

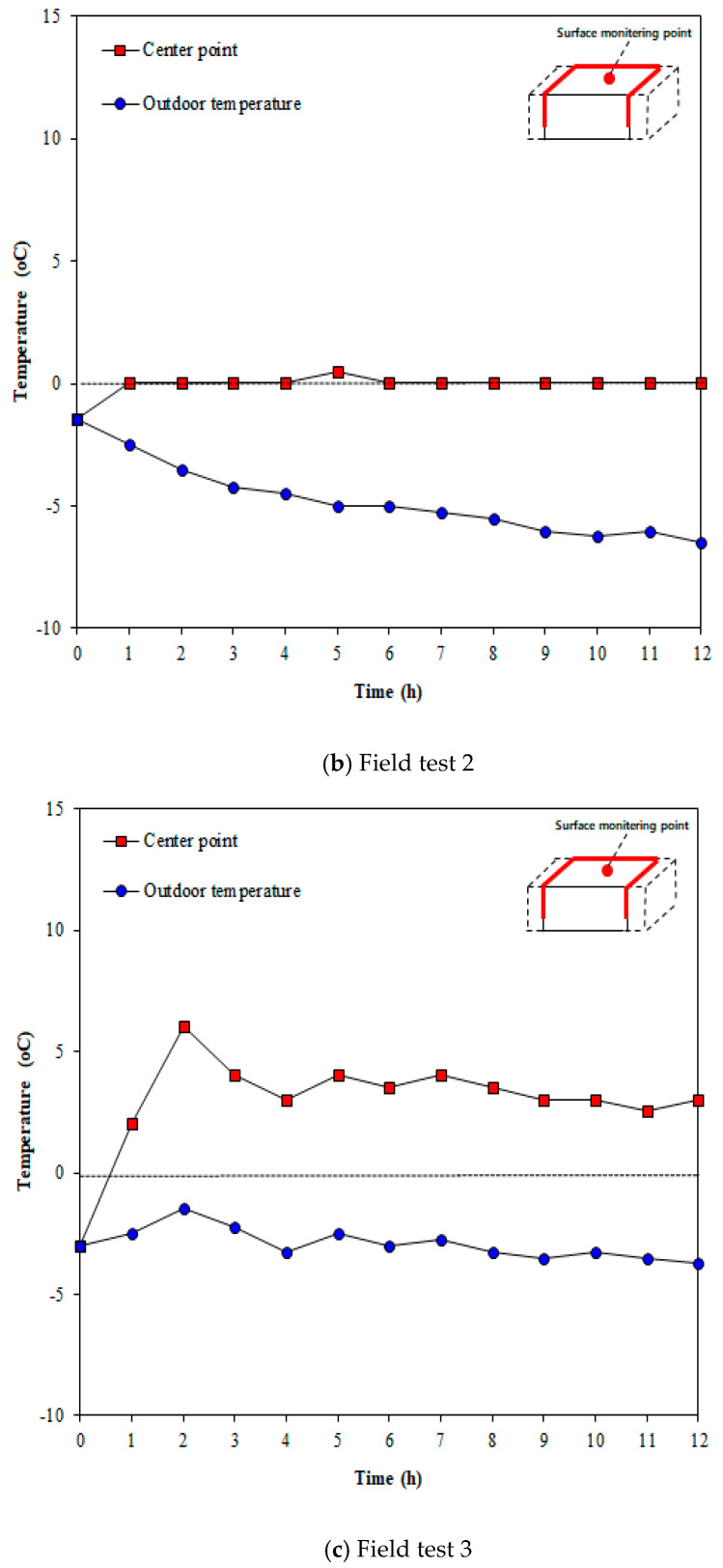
Temperature at center vs. outdoor temperature (Line 1).

The tests for verifying the surface temperature change due to thermal overlap were performed thrice. Figure 13 illustrates the concrete surface temperatures measured in Line 1, which was expected to show the highest thermal overlap effect when the heating elements were arranged in an inverted S-shaped layout. Figure 13 shows the change in surface temperature over time. The temperature was measured at 1 h intervals for 12 h after the test started, but only the measurements at 2 and 12 h were compared for convenience. As shown in the figure, the surface temperature increases over time. Figure 14 presents the comparison of temperatures between the center point of concrete and outdoor temperature. As shown in Figure 14a,b, the outdoor temperature kept decreasing over the time; nonetheless, the surface temperature of concrete was slightly increased. According to Figure 14c, the outdoor temperature increases until 2 h after the test started; consequently, the surface temperature increases until 2 h after the test started. Furthermore, 12 h after the test started, the surface temperature at the center was higher by 6.4 °C on average than the outdoor temperature.

**Figure 15:**
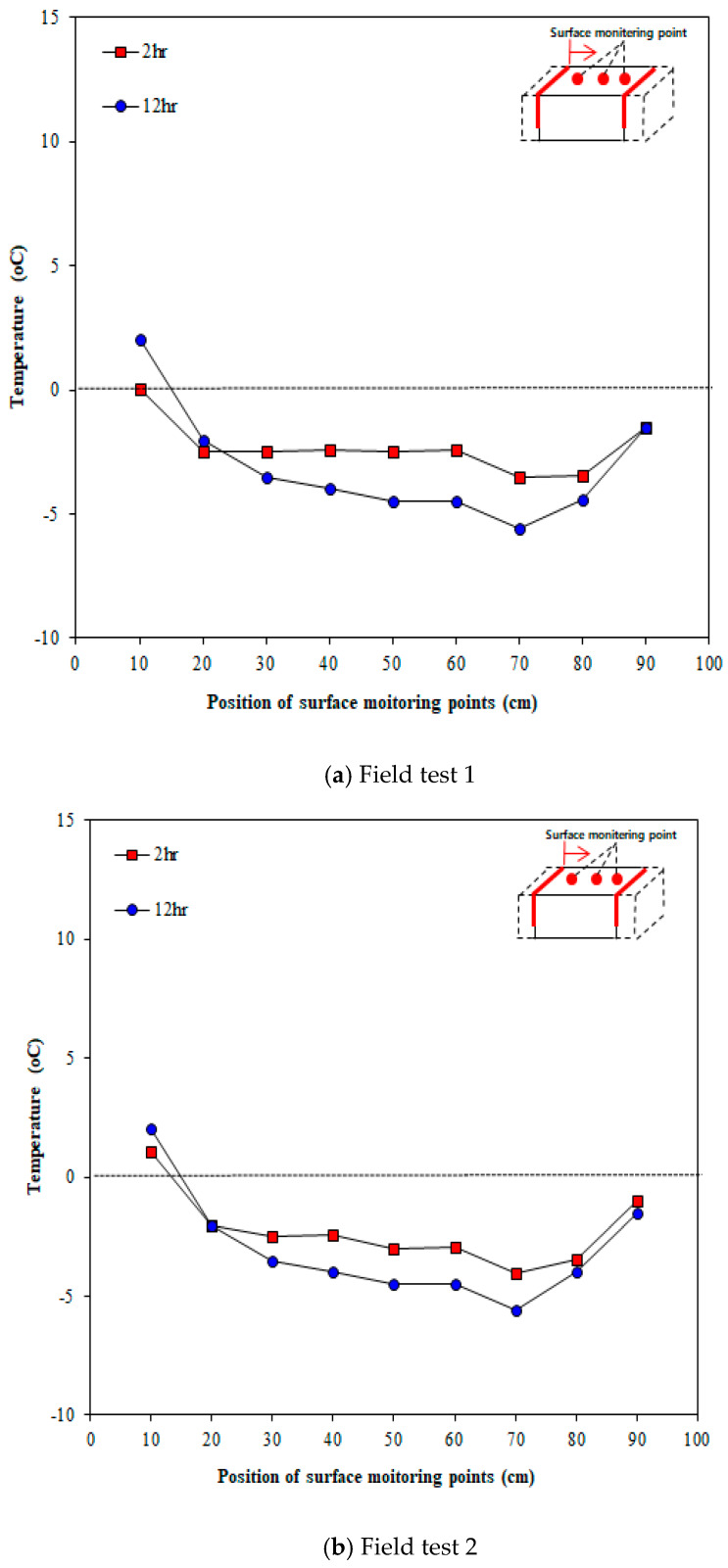

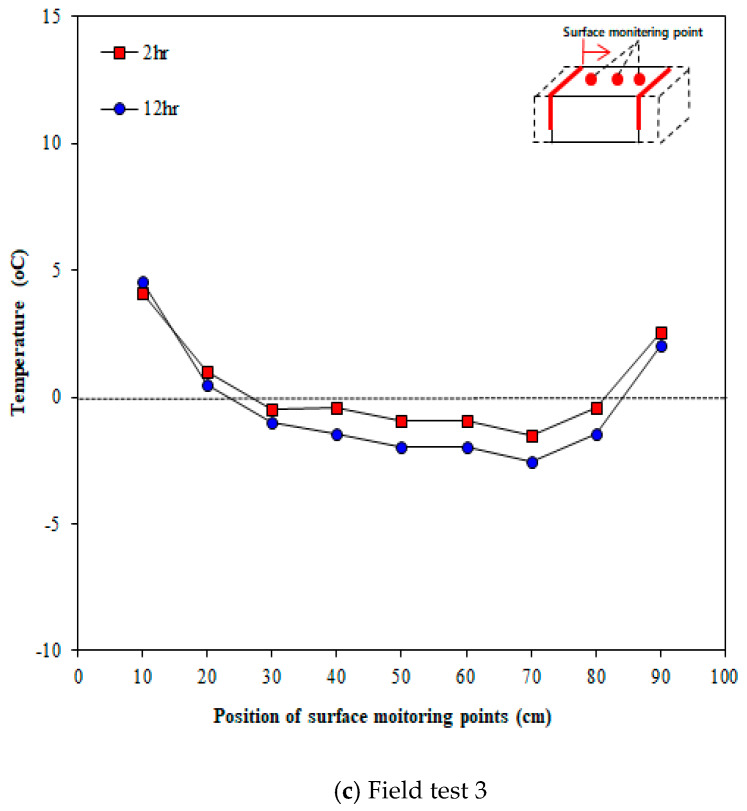
Temperatures at different surface locations measured at different times (Line 2).

**Figure 16:**
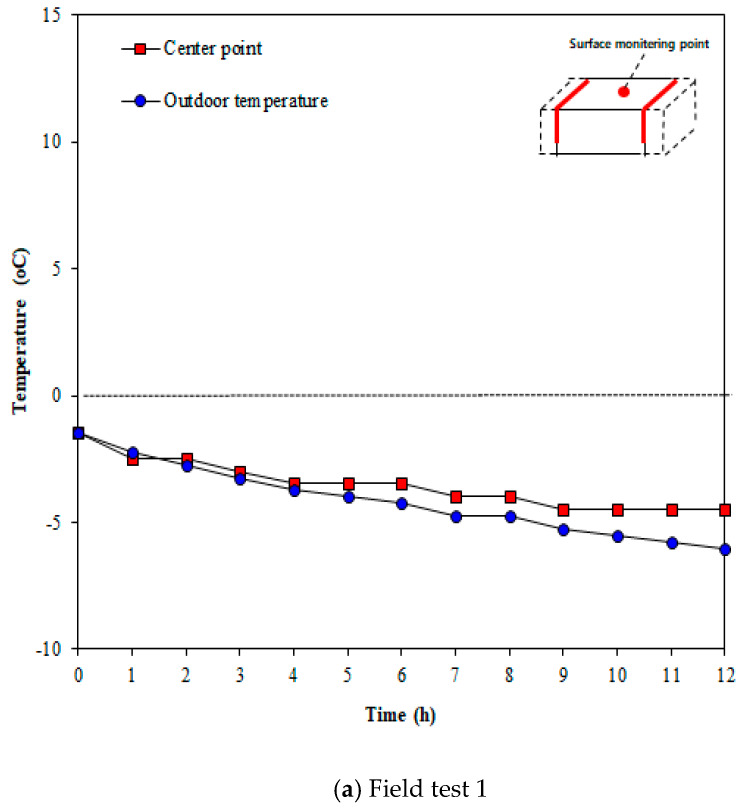

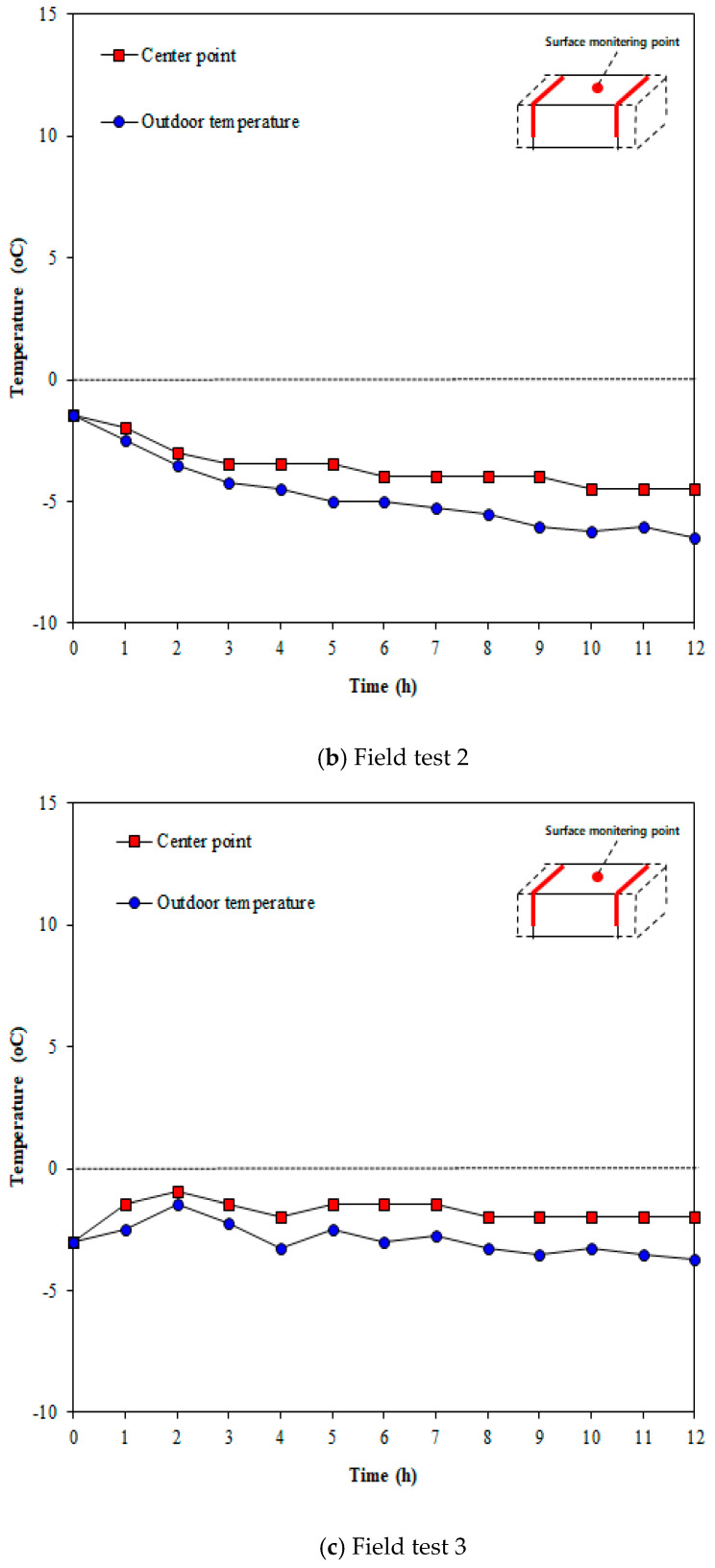
Temperature at center vs. outdoor temperature (Line 2).

Figure 15 illustrates the concrete surface temperatures measured in line 2, which was expected to show the smallest thermal overlap effect when the heating elements were installed in an inverted S-shaped layout. As shown in the figure, the surface temperature changes over time. The surface temperature decreased over time, unlike line 1. This suggests that the surface temperature is low, owing to the low thermal overlap effect because the overlapping part of the heating elements was small plus outdoor temperature was decreased over time. Figure 16 presents the comparison of temperatures between the center point of concrete and outdoor temperature. As shown in the figure, the surface temperature of concrete was clearly high compared to the outdoor temperature but was not sufficient to go above zero. This is because the overlapping effect was small and the outdoor temperature kept lowering over time.

**Figure 17:**
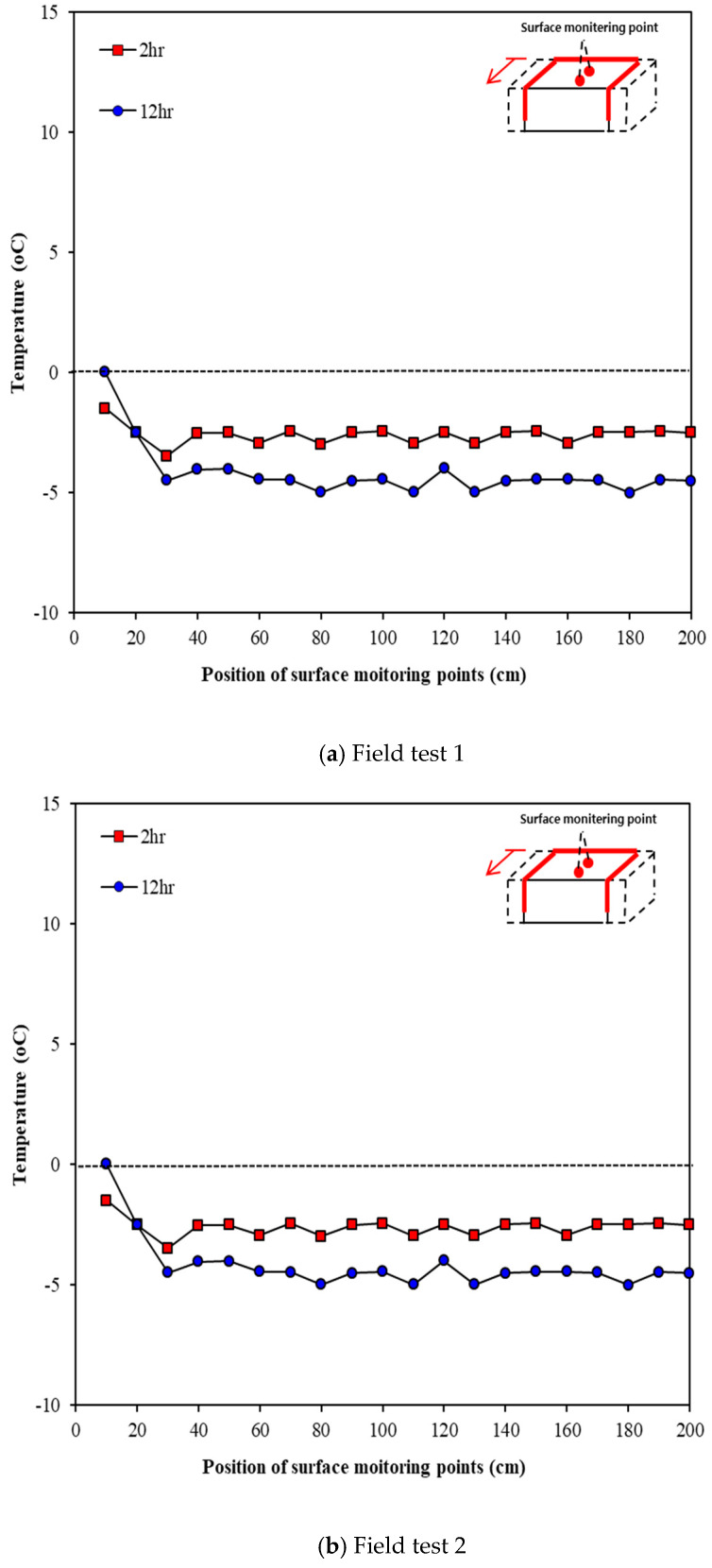

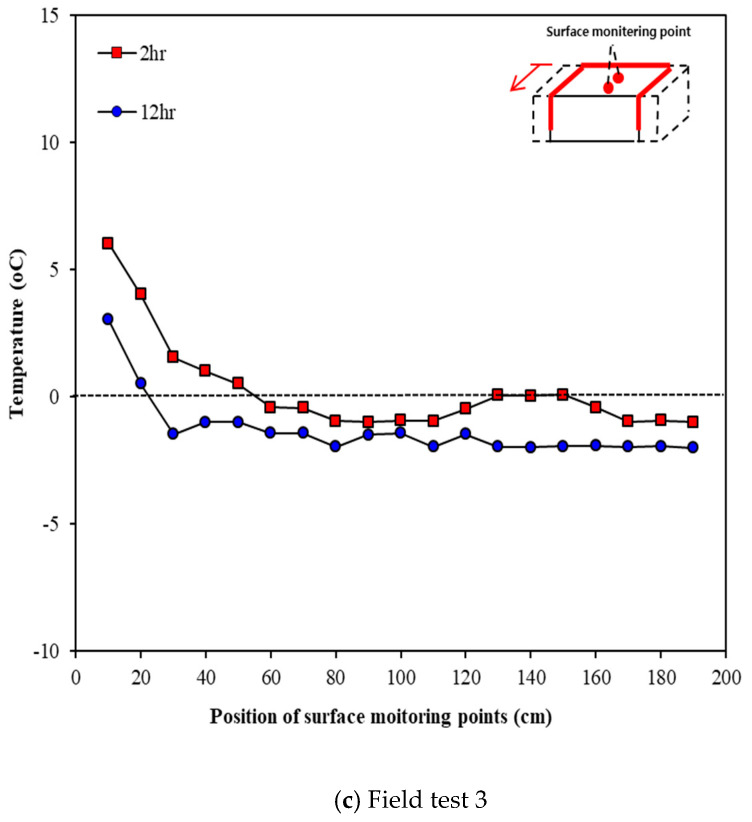
Variations in temperatures at different surface locations with time (Line 3).

We measured the temperature in line 3 to verify the temperature change at the center between the heating elements when the heating elements were installed in the inverted S-shaped layout. Figure 17 shows that the surface temperature decreases over time as the outdoor temperature decreases. However, the surface temperature increased by 2 °C on average compared to the outdoor temperature at locations 30 to 200 cm from the heating element, where the thermal overlap effect reduced.

**Figure 18:**
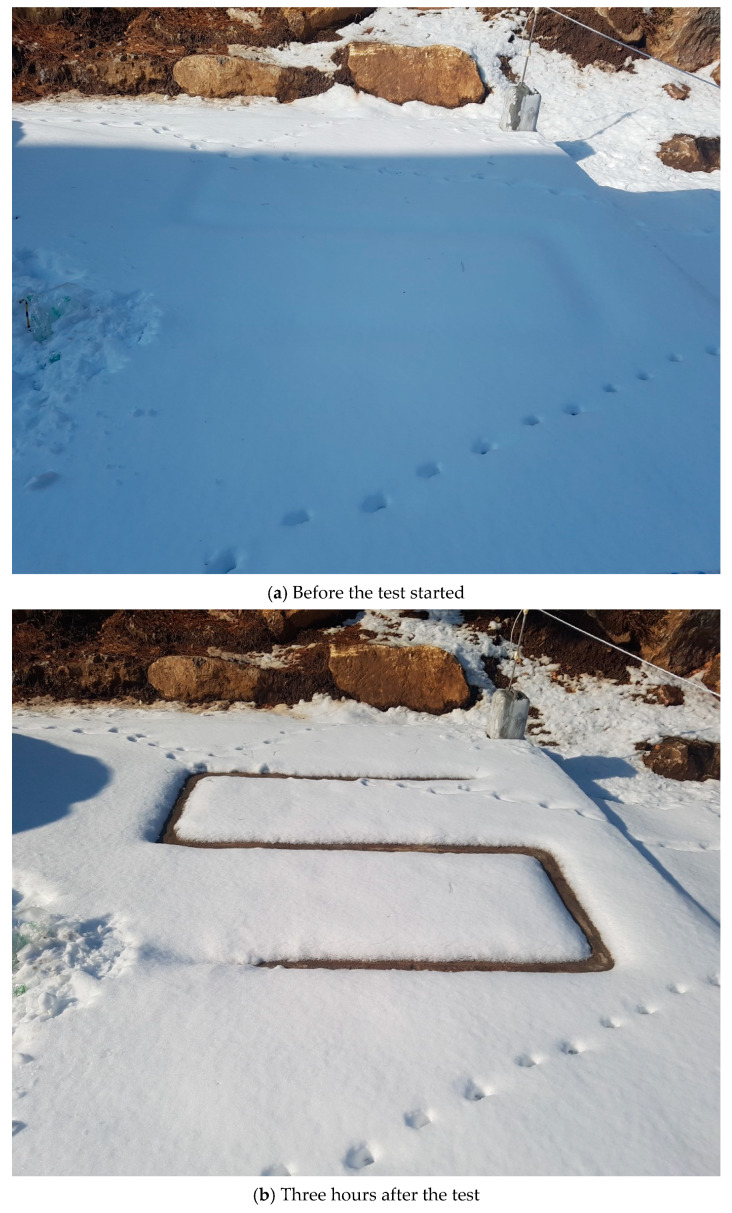

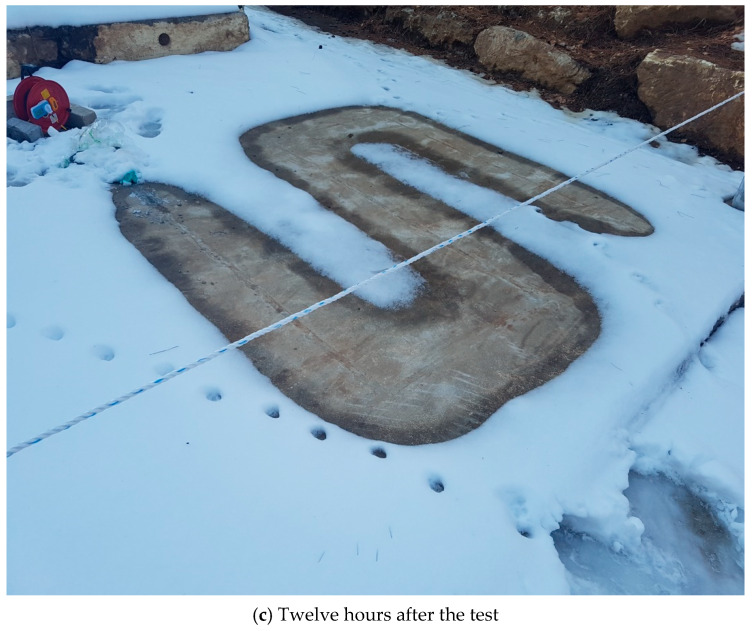
Deicing after snow accumulation.

To determine the possibility of deicing by melting snow, a field test was performed by operating the heating elements when the snowfall depth was 6.5 cm, as shown in Figure 18a. Figure 18b shows a photograph taken 3 h after the test started, and the width of the melted part was 10 cm. Figure 18c shows a photograph taken 12 h after the test started, and the distance of the melted part was 62 cm. Therefore, deicing is possible when heating elements are installed in an inverted S-shaped layout on the road. Furthermore, as there was no traffic during the field tests, the deicing effect is expected to be greater on actual roads than that observed during the field test.

## 5. Conclusions

Indoor experiments, numerical analyses, and field tests were conducted to develop a deicing technology that can be used in existing roads as well as new roads. The following conclusions were drawn from the findings:

1. When one heating element was used in the indoor experiment, the effective heating distance (distance from the heating element to the point where the surface temperature is 0 °C) was 8 cm. Furthermore, the temperature at 15 cm from the heating element was −7.5 °C, higher by 2.5 °C than the initial sample temperature and ambient temperature (−10 °C). Therefore, the heat generated by the heating elements was transferred to the entire concrete sample.
2. When the heating elements were spaced at 15 and 20 cm, the surface temperature between the heating elements exceeded 0 °C. In contrast, when spaced at 30 cm, the temperature at the center (15 cm from the heating element) of the two heating elements was lower than 0 °C. However, the average surface temperature at the center between the two heating elements was −5 °C, higher by 2.5 °C than that observed when using a single heating element at the same distance. Therefore, although the thermal overlap effect at a spacing of 30 cm was not prominent (surface temperature higher than 0 °C), owing to the wide spacing between the heating elements, heat was transferred to the entire sample. These results of the indoor experiments show that the effective heating distance of the heating elements should be 20–30 cm.
3. During numerical analysis, the thermal conductivity values of the heating elements were determined based on the results of the indoor experiments. Based on these property values, numerical analysis was performed by increasing the heating element temperature to 100 °C and experiment time to 3 h with the heating elements spaced at 100, 150, and 200 cm. Consequently, the surface temperature was higher than 0 °C when the heating elements were spaced at 100 cm, but lower than 0 °C when spaced at 150 and 200 cm. These results indicate that the effective heating distance increases to 100 cm when the heating element temperature and experiment time are increased. In the field tests, the heating elements were spaced at 100 cm in an inverted S-shaped layout based on the numerical analysis results. The temperatures were measured in three areas according to the thermal overlap effect. In line 1, the thermal overlap effect was high, owing to the overlapping heating elements. Therefore, even when the outdoor temperature decreased over time, the temperature at the center measured 12 h after the test increased by 6.4 °C, compared to the outdoor temperature.
4. In contrast, in line 2, where the thermal overlap effect of the heating elements was small, the temperature at the center at 12 h after the test started increased by only 1.8 °C compared to the outdoor temperature.
5. The temperatures in line 3 were measured to verify the temperature changes at the center between the heating elements (50 cm from the heating elements). We found that the temperatures 30–200 cm from the heating element were similar to the center temperature in line 2. This suggests that the temperature was high up to 30 cm from the heating element, owing to the thermal overlap effect, but it converged to a constant value beyond 30 cm.

The results of the field tests performed to verify the possibility of deicing by melting snow showed that snow with a depth of 62 cm melted between the heating elements (100 cm), thus verifying the possibility of deicing. Furthermore, as there was no traffic during the field tests, the deicing effect on actual roads is expected to be greater than that observed during the field tests.

## Figures and Tables

**Table 1:** Concrete pavement mixing proportion.

Concrete Pavement Mixing Proportion (1000 L, kg/m^3^)	
Maximum size of coarse aggregate (mm)	25
W/C (%)	45
S/a (%)	37
Water (kg)	147
Cement (kg)	326
Fine aggregate (kg)	707
Coarse aggregate (kg)	1184
Air-entraining agent (kg)	1.956
Superplasticizer (kg)	1.956

* Specific gravity: cement (3.15), fine aggregate (2.66), coarse aggregate (2.73).

**Table 2:** Features of CNT.

Diameter (nm)	Density $\left(g/cm^{3}\right)$	Electric Resistance (Ωcm)	Current Density $\left(A/cm^{2}\right)$	Tensile Strength (GPa)	Thermal Conductivity (W/mK)
5–100	1.33–1.4	1.0 × 10^−6^	1.0 × 10^8^ ~ 1.0 × 10^9^	50–300	3000

**Table 3:** Physical properties of concrete.

References	Thermal Conductivity	Specific Heat	Density
[14]	0.86 W/m·°C	1046 J/kg·K	2600 kg/$m^{3}$
[15]	2.42 W/m·°C	1090 J/kg·°C	2400 kg/$m^{3}$

**Table 4:** iButton specifications.

Shape	Temp Range	Size
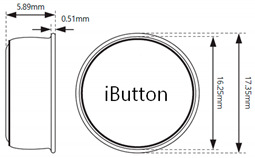	−40–85 °C	1.7 × 1.7 × 0.5 cm
